# Supplementary material for: Causal Relationship between Aging and Anorexia Nervosa: A White-Matter-Microstructure-Mediated Mendelian Randomization Analysis
Source: Biomedicines. 2024 Aug 16;12(8):1874. doi: 10.3390/biomedicines12081874 (PMC11351342; doi:10.3390/biomedicines12081874)
Supplement: Supplementary file 1 [file biomedicines-12-01874-s001.zip › Supplementary Figure/Figure S8/Leaveoneout plot of MR analysis for TL to ubm-b-2096.pdf]

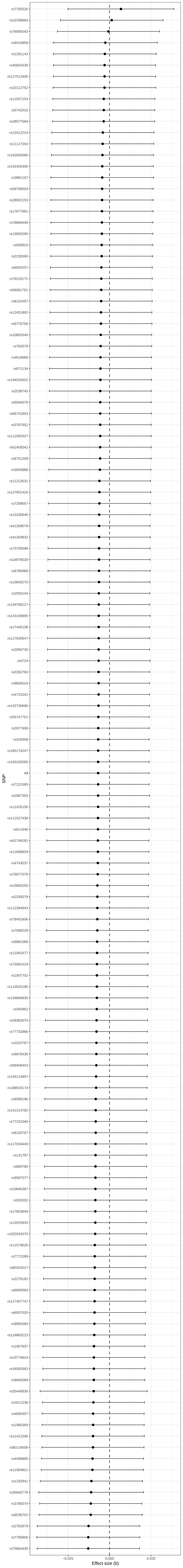
SNP
Effect size (b)
-0.025
0.000
0.025
rs7705526
rs10768683
rs76065543
rs9419958
rs1291143
rs45604339
rs117512405
rs10112752
rs11557154
rs5742915
rs28577594
rs12412214
rs11117354
rs182059586
rs142426306
rs3891167
rs56799554
rs28502153
rs17677991
rs76666449
rs13062095
rs939916
rs2230590
rs6054257
rs76219171
rs56061761
rs8102497
rs12451892
rs6776756
rs10805346
rs762679
rs4616688
rs871134
rs144204502
rs2538745
rs6584579
rs66731853
rs3767952
rs111950327
rs61405042
rs6751209
rs3093888
rs11212631
rs137901416
rs7209057
rs13230646
rs41269079
rs41304832
rs73730598
rs16978028
rs6790988
rs10840270
rs2555104
rs139795227
rs143190905
rs17445108
rs117630647
rs2056726
rs4724
rs2282764
rs9600019
rs4731541
rs142730696
rs55747751
rs2977608
rs429358
rs185174247
rs150150565
A8
rs7221585
rs2967355
rs11426156
rs111527438
rs611646
rs61748181
rs11699829
rs4743037
rs79977579
rs10905255
rs2293579
rs112394943
rs78491606
rs7099229
rs6881568
rs11991877
rs73581419
rs1907702
rs113525195
rs139669835
rs450962
rs28363070
rs77732866
rs1023767
rs9878436
rs59409453
rs145114957
rs188918174
rs9398196
rs141214782
rs77231040
rs8105767
rs117034449
rs131797
rs869785
rs6587577
rs10845387
rs932002
rs17803849
rs12925933
rs202034370
rs11579626
rs7772289
rs80324517
rs2276182
rs6669563
rs117407747
rs6007020
rs9955360
rs116863223
rs1957937
rs10774624
rs34550383
rs9940099
rs35446936
rs1611236
rs4695407
rs1985369
rs11412296
rs80116508
rs4498805
rs11584821
rs1332941
rs35640778
rs3785074
rs6536702
rs2763979
rs7790856
rs75664430
